# Improving real-world evaluation of patient- and physician-reported tolerability: niraparib for recurrent ovarian cancer (NiQoLe)

**DOI:** 10.1093/jncics/pkae114

**Published:** 2024-12-14

**Authors:** Florence Joly, Fernando Bazan, Delphine Garbay, Yaelle Ouldbey, Philippe Follana, Élise Champeaux-Orange, Eric Legouffe, Pierre-Emmanuel Brachet, Dominique Spaeth, Pierre Combe, Anne-Claire Hardy-Bessard, Frédéric Selle, Julien Grenier, Coriolan Lebreton, Olfa Derbel, Elise Bonnet, Pierre Fournel, Yolanda Fernandez Diez, Valérie Delecroix, Sheik Emambux, Jérôme Alexandre, Thomas Grellety, Dominique Mille, Hubert Orfeuvre, Catherine Favier, Delphine Le Roux, Marie-Ange Mouret-Reynier, Stanislas Quesada, Jean-Emmanuel Kurtz

**Affiliations:** Department of Medical Oncology, Centre François Baclesse, University Unicaen, Caen, France; Department of Oncology, CHRU Besançon—Hôpital Jean Minjoz, Besançon, France; Medical Oncology Department, Clinique Tivoli-Ducos, Bordeaux, France; Department of Clinical Research and Innovation, Centre Léon-Bérard, Lyon, France; Department of Medical Oncology, Centre Anticancer Antoine Lacassagne, Nice, France; Medical Oncology Department, CHU d’Orléans, Orléans, France; Oncology Department, Oncogard—Polyclinique KenVal Institut de Cancérologie du Gard, Nimes, France; Department of Medical Oncology, Centre François Baclesse, University Unicaen, Caen, France; Medical Oncology Department, Centre d’Oncologie de Gentilly, Nancy, France; Department of Medical Oncology, Pôle Santé Léonard de Vinci, Chambray-Lès-Tours, France; Department of Medical Oncology, Centre Armoricain d’Oncologie, Plérin, France; Department of Medical Oncology, Groupe Hospitalier Diaconesses Croix Saint-Simon, Paris, France; Department of Medical Oncology, Institut du Cancer Avignon Provence, Avignon, France; Department of Medical Oncology, Institut Bergonié, Bordeaux, France; Department of Medical Oncology, Centre Léon-Bérard, Lyon, France; Medical Oncology Department, Groupe Hospitalier Mutualiste (GHM) de Grenoble, Grenoble, France; Department of Medical Oncology, Nord University Hospital, Saint Etienne, France; Medical Oncology Department, Institut de Cancérologie de Lorraine (ICL), Vandoeuvre-Lès-Nancy, France; Department of Medical Oncology, Clinique Mutualiste de l’Estuaire, Saint-Nazaire, France; Medical Oncology Department, CHU de Poitiers—Hôpital de la Milétrie, Poitiers, France; Université Paris Cité, APHP, Department of Medical Oncology, Hôpital Cochin, Paris, France; Medical Oncology Department, Centre Hospitalier de la Côte Basque, Bayonne, France; Department of Medical Oncology, Médipôle de Savoie, Challes-les-Eaux, France; Medical Oncology Service, Fleyriat Hospital Center, Bourg en Bresse, France; Department of Medical Oncology, Centre Georges-François Leclerc, Dijon, France; Department of Medical Oncology, Saint Malo Hospital, Saint Malo, France; Department of Medical Oncology, Centre Jean-Perrin, Clermont-Ferrand, France; Medical Oncology Department, Montpellier Cancer Institute (ICM), Montpellier, France; Department of Medical and Surgical Oncology and Hematology, Institut of Cancer Strasbourg (ICANS), Strasbourg, France

## Abstract

**Background:**

Maintenance niraparib at an individualized starting dose (ISD) is established in platinum-sensitive recurrent ovarian cancer (PSROC). However, patients’ perspectives on the burden of prolonged maintenance therapy have not been reported in prospective trials or routine practice.

**Methods:**

In the real-life multicenter NiQoLe study, patients with PSROC received ISD maintenance niraparib. The primary objective was to describe physician-reported adverse events (AEs) leading to treatment modification during the first 3 months. Secondary endpoints included patient-reported outcomes (symptomatic AEs using PRO-CTCAE, self-reported fatigue, and impact on daily activities/function using FACT-F) collected remotely weekly using a specifically designed electronic device.

**Results:**

Most (80%) of 139 treated patients (median age = 70 years) began niraparib at 200 mg/day. Median treatment duration was 5.7 (range = 0.2-21.4) months. During the first 3 months, 86 patients (62%) required treatment modification (median = 27 days to modification). Physician-reported grade ≥3 niraparib-related AEs occurred in 34 patients (24%); 68 patients (49%) had treatment modification for AEs, predominantly thrombocytopenia. The most frequent patient-reported AEs (PRO-CTCAE) were fatigue, insomnia, constipation, and dry mouth. Self-reported AEs were severe in 66% of patients. At baseline, 33% of patients reported severe fatigue (FACT-F), which generally persisted during niraparib. Physicians systematically underestimated major patient-reported symptoms.

**Conclusions:**

In routine practice, niraparib dose modification was often required during the first 3 months despite individualized dosing. Physicians underestimated the burden of fatigue and symptomatic AEs. Digital self-reporting of AEs is feasible, provides patient-centered information complementing physician-reported AEs, and allows fuller appreciation of toxicity in real-world studies.

**Clinical trial information:**

NCT03752216

Platinum-based therapy is standard at diagnosis of ovarian cancer (OC) and at relapse if it occurs 6 months or more after completing frontline therapy. Patients responding to platinum rechallenge receive maintenance therapy with a poly(ADP-ribose) polymerase inhibitor (PARPi), such as niraparib.^1-4^

The efficacy and safety of maintenance niraparib has been demonstrated in several phase 3 trials.^3^^,^^5^^,^^6^ In NOVA (in late-relapsing recurrent OC), the most common adverse events (AEs) were gastrointestinal and hematological effects, fatigue, headache, and insomnia.^3^ Subsequent trials established an individualized starting dose tailored according to baseline weight and platelet count, offering improved tolerability while maintaining efficacy.^6-9^ However, retrospective real-world data suggest more frequent dose modifications and treatment discontinuation for AEs in unselected populations treated in routine practice than reported in pivotal trials.^10^ Prospective clinical trials extensively describe physician-reported AEs but provide minimal information on patient-reported toxicities. Furthermore, retrospective real-world studies rely on physician-documented AEs,^11^ and none has used the Patient-Reported Outcomes version of the Common Terminology Criteria for Adverse Events (PRO-CTCAE), which characterizes symptomatic treatment toxicities from the patient’s perspective.^12^

PRO analyses from NOVA and PRIMA indicated maintained health-related quality of life (HRQoL) during maintenance niraparib,^13^^,^^14^ but the questionnaires used did not capture PARPi-specific side effects (eg, fatigue), patient-reported AEs, or the impact of persistent low-grade AEs during prolonged maintenance therapy. Chronic side effects promote “pill fatigue” and may affect treatment compliance.^15^

Patients may hesitate to report symptoms because of time constraints, fear of stopping treatment, or difficulty remembering symptoms between clinic visits.^16^ Physicians may focus on expected AEs, objective signs, or asymptomatic AEs with a direct medical impact (eg, platelet count, liver function tests); they may have limited time or may not systematically ask about symptoms. Missed or delayed symptom detection can lead to suboptimal treatment modification, detrimentally affecting treatment adherence, symptom control, and HRQoL. However, many of these challenges can be overcome if PROs are collected digitally at home and accessed by the care team to adapt patient management. Understanding treatment burden is particularly important in the maintenance setting, where disease-related symptoms are less bothersome and acceptance of treatment-related AEs may be lower.

To explore the impact of maintenance niraparib on patients with OC treated in routine practice, we initiated the real-world Niraparib and Quality of Life (NiQoLe) study, integrating digitally collected patient-reported AE monitoring into the study design.

## Patients and methods

NiQoLe (GINECO-OV239b; NCT03752216) was an open-label longitudinal real-world study conducted at 27 French sites. Eligible patients had high-grade epithelial OC and a complete or partial response after completing platinum-based chemotherapy less than 12 weeks before initiating maintenance niraparib. Patients with grade 3/4 anemia, neutropenia, or thrombocytopenia related to their last chemotherapy and persisting for more than 4 weeks were ineligible.

Maintenance oral niraparib was started at 300 mg daily, or 200 mg daily in patients with baseline body weight less than 77 kg and/or platelet count less than 150 000/μL. The daily dose was reduced by 100 mg in the case of AEs. Each cycle lasted 28 days, and maintenance therapy was continued until disease progression or unacceptable toxicity.

The primary objective was to evaluate the incidence, grade (National Cancer Institute CTCAE version 5.0), and type of AEs leading to dose modification during the first 3 months of maintenance niraparib, as reported by physicians. Secondary objectives reported here included evaluation of patient-reported fatigue (assessed by Functional Assessment of Cancer Therapy-Fatigue [FACT-F]^17^) and other symptoms and side effects, including fatigue (PRO-CTCAE version 1.0^12^); side-effect management and reasons for dose modifications; patient-reported treatment adherence; time to onset and duration of AEs; and treatment duration. Additionally, we explored concordance between physician- and patient-reported effects.

Every week during the first 6 months and every 3 months thereafter, patients reported selected symptomatic AEs (PRO-CTCAE, grades 0-3), fatigue (FACT-F), and treatment compliance remotely using a handheld electronic device linked to a first-generation computer-based health evaluation system (CHES;^18^  Figure S1). To reduce patient burden, only the most relevant PRO-CTCAE items were selected, in accordance with European Society for Medical Oncology guidelines.^16^ Frequency, severity, and interference were reported individually (if assessed), and composite grades ranging from 0 to 3 were calculated according to previously described methods^12^^,^^19^ (Supplementary Methods and Tables S1 and S2). Clinical trial monitors communicated PRO-CTCAE and FACT-F data to physicians every month and before each scheduled follow-up visit. Data on treatment compliance, tolerability, treatment modifications, physician-reported side-effect management, and physician-reported AEs were collected every 3 months. Clinical progression was assessed at the same timepoints according to RECIST, version 1.1. Complete blood count assessment was undertaken at baseline and then at 3, 6, 12, and 18 months after starting niraparib.

### Statistical analysis

No formal statistical testing was planned in this open-label real-world trial in routine practice, and all analyses were descriptive. Safety and progression-free survival (PFS) were analyzed in all patients who received at least 1 dose of niraparib. HRQoL was analyzed in all treated patients with a baseline HRQoL assessment.

A 3-point minimal important difference was used to classify each weekly FACT-F score as worsened, stable, or improved. The most frequent classification was reported as the overall score. The proportion of patients with severe fatigue (FACT-F score ≤37^20^^,^^21^) was calculated at each timepoint.

All patients provided signed informed consent before undergoing any study-specific procedures. The study was performed according to the ethical principles of the Declaration of Helsinki and the applicable International Conference for Harmonisation Good Clinical Practice regulatory requirements. The study protocol and informed consent forms were approved by the Comité de Protection des Personnes (CPP) Sud Est II.

## Results

Between April 11, 2019, and May 18, 2021, 141 patients were enrolled, of whom 139 received niraparib (1 withdrew consent and 1 experienced disease progression before starting niraparib). Few patients (<10%) had *BRCA1/2*-mutated disease (Table 1). More than half were older than 70 years of age; among the 55 patients with baseline oncogeriatric information, 35 (64%) had a Geriatric G8 score higher than 14. The database lock was December 15, 2022 (December 5, 2022, for the CHES data).

**Table 1.** Baseline characteristics

Characteristic	Treated population (N = 139)
Median [range] age, years	70 [44-88]
Age >70 years, n (%)	75 (54)
ECOG performance status, n (%)	
0	70 (50)
1	67 (48)
2	2 (1)
FIGO stage, n (%)^a^	
I–IIIA	17 (13)
IIIB	16 (13)
IIIC	70 (56)
IV	23 (18)
Histology, n (%)	
High-grade serous	127 (91)
Grade 2/3 endometrioid	5 (4)
Undifferentiated	5 (4)
Other	2 (1)
*BRCA1* or *BRCA2* deleterious mutation, n (%)^b^	7 (7)
Weight <77 kg, n (%)	103 (74)
Platelets <150 000/μL, n (%)	8 (6)
Surgery, n (%)	131 (94)
Residual disease after last surgery	49/131 (37)
No. of prior lines of platinum-based therapy before recurrence, n (%)	
1	106 (76)
2	27 (19)
3	2 (1)
4	2 (1)
5	2 (1)
Median [range]	1 [1-5]
Prior bevacizumab, n (%)	99 (71)
Prior olaparib, n (%)	5 (4)
Response to last platinum, n (%)	
Complete response	48 (35)
Partial response	78 (56)
Stable disease	13 (9)^c^
Median [range] interval between last platinum and niraparib, days	49 [15-109]

a FIGO status missing in 13 patients.

b *BRCA* mutation status missing in 34 patients. Among the 7 patients with tumors harboring a *BRCA1/2* mutation, 2 had germinal *BRCA1* mutation, 1 had somatic *BRCA1* mutation, 3 had germinal *BRCA2* mutation, and 1 had somatic *BRCA2* mutation (none had tumors harboring both *BRCA1* and *BRCA2* mutations).

c Ineligible per protocol but enrolled and treated in error.

Abbreviations: ECOG = Eastern Cooperative Oncology Group; FIGO = International Federation of Gynecology and Obstetrics.

### Treatment exposure and modification

Most patients (80%) started niraparib at 200 mg/day (Table 2). The median duration of niraparib was 5.7 (range 0.2–21.4) months; 63 patients (45%) continued treatment for ≥6 months. During the first 3 months, 86 patients (62%) had their treatment modified after a median of 27 days. Treatment was modified because of AEs in 68 patients (49%; Table 2 and Figure S2).

**Table 2.** Treatment modification during the first 3 months of niraparib

Modification	No. of patients (%)			Median [range] time to first modification, days (N = 139)
	Starting dose 300 mg/day (n = 27)	Starting dose 200 mg/day (n = 111)	**All patients (N = 139)** ^a^	
Any treatment modification	21 (78)	65 (59)	86 (62)	27 [0-90]^b^
Treatment discontinued for disease progression	4 (15)	19 (17)	23 (17)	70 [6-91]
Treatment modification for AE	17 (63)	51 (46)	68 (49)	25 [0-91]^b^
Dose reduced	13 (48)	34 (31)	47 (34)	42 [8-90]^c^
Treatment interrupted	11 (41)	42 (38)	53 (38)	25.5 [0-90]^b^
Treatment discontinued	5 (19)	7 (6)	12 (9)^d^	20.5 [6-91]
Treatment modification for patient convenience	1 (4)	5 (5)	6 (4)	37 [21-84]^e^
Dose reduced	1 (4)	1 (1)	2 (1)	33 [29-37]
Treatment interrupted	0	2 (2)	2 (1)	21 [21-21]^e^
Treatment discontinued	0	2 (2)	2 (1)	80.5 [77-84]
Treatment modification for other reason	4 (15)	1 (1)	5 (4)	47 [22-91]
Dose reduced	4 (15)	0	4 (3)^f^	51.5 [22-77]
Treatment interrupted	1 (4)	1 (1)	2 (1)^g^	62 [33-91]

a One patient who weighed ≥77 kg and had platelets ≥150 000/μL took a starting dose of 100 mg in error.

b Missing date of first dose interruption for AE (and, consequently, date of first dose modification and dose modification for AE) for 1 patient.

c Missing date of first dose reduction for AE for 1 patient (but not missing date of earlier treatment interruption for AE).

d Preceded by treatment interruption and dose reduction in 1 patient.

e Missing date of first dose interruption for patient convenience (and, consequently, date of first dose modification for patient convenience) for 1 patient.

f Described as investigator decision in 3 patients (also mentioning decreased platelet count for 1 patient) and general alteration in 1 patient.

g Described as investigator decision in 1 patient and suspicion of disease progression in 1 patient.

Abbreviation: AE = adverse event.

Niraparib was discontinued permanently within the first 3 months in 37 patients (27%): 23 (17%) because of disease progression, 12 (9%) for AEs, and 2 (1%) for patient convenience (Table 2). The first treatment modification was due to thrombocytopenia in 44 patients (65% of 68 patients with treatment modification for AEs, 32% of all treated patients), occurring at grade 4 in 8 patients, grade 3 in 8 patients, grade 2 in 21 patients, and grade 1 in 7 patients. In 8 (18%) of these 44 patients, thrombocytopenia recurred at the same or a higher grade within the first 3 months despite dose modification.

During the first 3 months, 95 (69%) of 137 responding patients reported never missing a dose, 15 (11%) reported missing 1 dose, and 27 (20%) missed more than 1 dose.

### Physician-reported AEs

**Figure 1.**
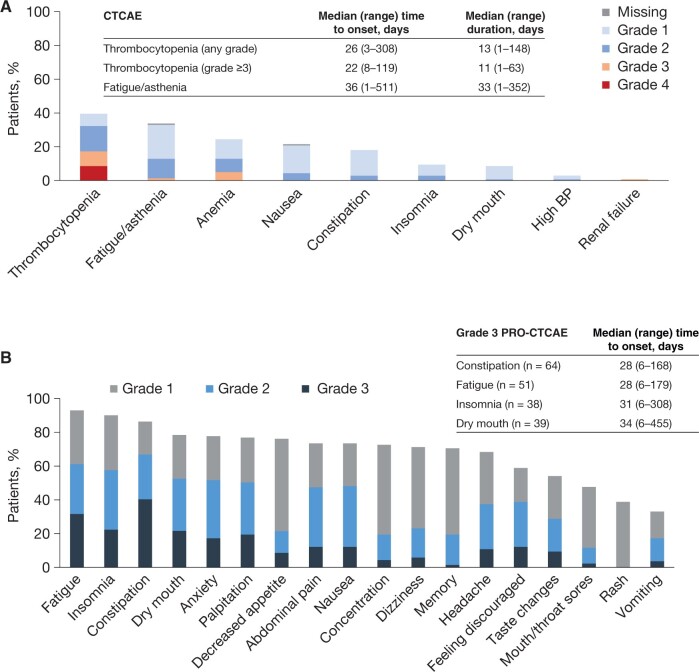
Summary of safety during the first 3 months. **A)** Physician-reported AEs. **B)** Patient-reported AEs (composite grade of 0-3 combining individual scores for frequency, severity, and interference with daily activities; grade not collected for rash). Abbreviations: AE = adverse event; BP = blood pressure; CTCAE = Common Terminology Criteria for Adverse Events; PRO-CTCAE = Patient-Reported Outcome version of the Common Terminology Criteria for Adverse Events.

Physicians reported grade 3 or higher AEs in 39 patients (28%) during the first 3 months, considered niraparib-related in 34 patients (24%). There was 1 fatal AE (treatment-related sepsis). The most common physician-reported AEs (any grade) were thrombocytopenia (40%) and fatigue/asthenia (34%), with median onset after approximately 1 month (Figure 1, A). The grade 3 or higher AEs most often reported by physicians were thrombocytopenia (17%; 9% grade 4) and anemia (5%; all grade 3).

### Patient-reported AEs

**Figure 2.**
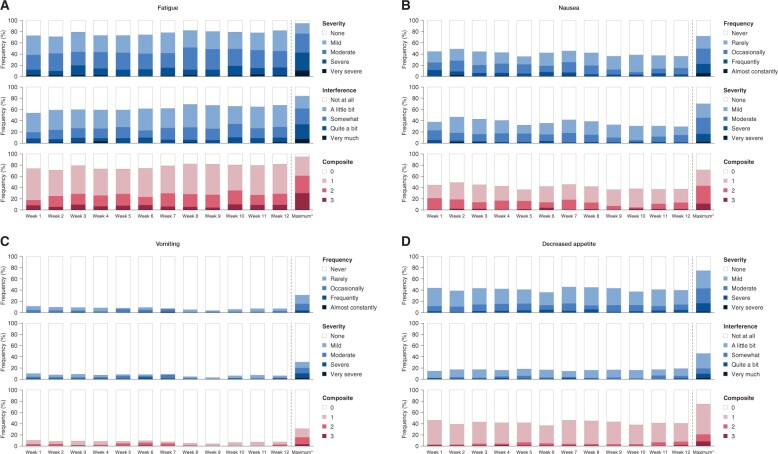
Individual item scores for severity, interference, and composite grade every week during the first 12 weeks of treatment and maximum post-baseline score.^19^  **A**) Fatigue (PRO-CTCAE fatigue, tiredness, or lack of energy). **B**) Nausea. **C**) Vomiting. **D**) Decreased appetite. Abbreviation: PRO-CTCAE = Patient-Reported Outcome version of the Common Terminology Criteria for Adverse Events. *Maximum score or grade reported postbaseline per patient.

Weekly PRO-CTCAEs were completed up to week 25 by ≥60% of patients (Figure S3). During the first 3 months, 98% of patients reported at least 1 symptomatic PRO-CTCAE (grade 3 in 66%). The most common PRO-CTCAEs were fatigue (93%; 32% grade 3), insomnia (90%; 22% grade 3), constipation (86%; 40% grade 3), and dry mouth (78%; 22% grade 3) (Figure 1, B). The composite score combining severity and interference of fatigue was high (grade 2/3) in approximately 20%-30% of patients each week during the first 3 months (Figure 2). The composite score combining frequency and severity of nausea was grade 2/3 in less than 20% and remained stable; grade 2/3 vomiting and decreased appetite were minimal throughout. Figure S4 shows other PRO-CTCAEs.

### Physician- vs patient-reported AEs

Gastrointestinal effects (nausea, constipation) were more than 3-fold more frequent in patient vs physician reporting (Table 3). Similarly, physicians reported fatigue in 34% of patients, whereas 93% of patients self-reported fatigue. The discrepancy was even more pronounced (up to 10-fold) for dry mouth and insomnia. The proportions of patients with (very) severe AEs were negligible by physician reporting but up to 40% self-reported by patients.

**Table 3.** Discrepancy between selected patient- and physician-reported AEs during the first 3 months of niraparib

AE	Patients with self-reported PRO-CTCAE, n (%) (N = 139)		Patients with physician-reported CTCAE, n (%) (N = 139)	
	Any grade	Severe (grade 3)	All	Severe (grade ≥3)
Fatigue	129 (93)	44 (32)	47 (34)	2 (1)
Nausea	102 (73)	17 (12)	30 (22)	0
Constipation	120 (86)	56 (40)	25 (18)	0
Dry mouth	109 (78)	30 (22)	12 (9)	0
Insomnia	125 (90)	31 (22)	13 (9)	0

Abbreviations: AE = adverse event; PRO-CTCAE = Patient-Reported Outcome version of the Common Terminology Criteria for Adverse Events.

### FACT-F

Thirty-five (33%) of 107 patients completing the FACT-F questionnaire reported severe fatigue at baseline. Although mean self-reported fatigue scores remained high (representing low fatigue) over time (Figure S5, A), the proportion of patients with severe fatigue (score ≤37) did not decrease. Patients with severe fatigue at baseline reported severe fatigue during the first 12 weeks at a higher proportion of timepoints than those without severe fatigue at baseline (mean = 72% vs 25% of timepoints). Fatigue worsened from baseline in 81 patients (76%), typically in the first 3 months with a median time to worsening of 0.9 months (95% confidence interval [CI] = 0.7 to 1.7) (Figure S5, B). In analyses categorizing FACT-F scores as worsened, stable, or improved during the first 3 months, 52 (49%) of 106 patients with at least 1 postbaseline questionnaire most often reported worsening, 24 (23%) reported a predominantly unchanged fatigue score, and 30 (28%) most frequently reported an improvement. FACT-F scores were available before and after progression (±3 months) for 41 patients. Comparison of pre- and post-progression scores revealed a deterioration in fatigue in 9 patients (22%), stable fatigue in 19 patients (46%), and an improvement in 13 patients (32%).

### Efficacy

At the data cutoff date, median follow-up for efficacy was 17.9 (95% CI = 17.6 to 19.1) months. PFS events had been recorded in 111 patients (80%); 67 (48%) had died. The median PFS was 6.2 (95% CI = 5.5 to 8.2) months. The estimated 3-month PFS rate was 81% (95% CI = 74% to 87%). In the subgroup of 126 patients with a complete or partial response to the most recent platinum-based therapy (ie, excluding those with stable disease after platinum therapy, who did not meet the eligibility criteria for NiQoLe), median PFS was 6.7 (95% CI = 5.5 to 8.3) months and the estimated 3-month PFS rate was 82% (95% CI = 74% to 87%).

## Discussion

The need for meaningful PRO reporting and evaluation in clinical trials has long been recognized, and approaches to improve reporting continue to evolve.^22^^,^^23^ The COVID-19 pandemic triggered rapid development and implementation of remote reporting, and recent guidelines recommend digital symptom monitoring (eg, with a handheld device) in routine care during systemic cancer treatment.^16^ However, when the NiQoLe study was initiated, integrating self-reported AEs and fatigue into safety monitoring was a groundbreaking approach. To our best knowledge, this is the first study to provide prospective longitudinal evidence on the burden of maintenance PARPi use (particularly fatigue) on patients with late-relapsing OC. A study strength is the remote self-reporting of symptomatic AEs using a handheld digital device, allowing frequent and regular reporting. Furthermore, results reflect the real-world experiences of a broader population than is typically enrolled in randomized clinical trials. Prospective data collection contrasts with the retrospective real-world reports in the literature.^10^^,^^11^^,^^24-27^ The NiQoLe study demonstrates the feasibility of digital self-reporting in routine practice. Although more than half the study population was older than 70 years of age, there was high compliance with PRO-CTCAE and FACT-F reporting, and patients coped well with digital data entry. Analyses focusing on patients older than 70 years are ongoing.

NiQoLe aimed to elucidate the trajectory of fatigue during maintenance therapy via intensive collection of PRO-CTCAEs and a fatigue-specific HRQoL questionnaire (FACT-F). As anticipated in this elderly study population, fatigue was particularly troublesome. Before starting niraparib, one-third of patients reported severe fatigue on FACT-F, highlighting the burden of chemotherapy and disease. The high level of fatigue persisted during maintenance niraparib, despite dose modification.

Most patients started niraparib at 200 mg. Nevertheless, 62% required niraparib treatment modification during the first 3 months, and thrombocytopenia was common. Careful monitoring is critical, especially during early cycles, to ensure dosing is truly individualized. Interestingly, although treating physicians were informed of PRO-CTCAEs, dose modifications were typically attributed to thrombocytopenia rather than symptomatic AEs, and a second modification was usually required. The need for further dose adjustment in patients receiving an individualized starting dose of niraparib is consistent with previous real-world studies of niraparib.^11^^,^^24^^,^^25^

The simple PRO-CTCAE method implemented in NiQoLe collects complementary information on the side effects of greatest relevance to patients receiving PARPi, allowing better treatment monitoring. Weekly remote reporting may improve information collection in the week after treatment initiation, which often coincides with the greatest symptom burden. For example, in the NOVA trial, PRO questionnaires were administered every 8 weeks, reflecting patients’ experience in the preceding 7 days,^13^ and yet most AEs and grade 3 or higher hematological and symptomatic AEs occurred during the first month of niraparib treatment and declined thereafter.^28^ The NOVA investigators reported a decrease over time in the proportion of patients experiencing lack of energy or fatigue, and no negative effect of hematological AEs on HRQoL. However, real effects may go undetected with relatively infrequent PRO data collection. In the NiQoLe study, PRO-CTCAEs were collected weekly during the first 3 months, when AEs are typically most frequent and burdensome.^28^ The median time to onset of the most common grade 3 PRO-CTCAEs was 28-34 days. Fatigue and nausea persisted at a similar severity/interference level over time, whereas severe/frequent vomiting and decreased appetite were less common. The NiQoLe design is in line with recommendations to match quality-of-life assessments to hypothesized symptom trajectories,^29^ perhaps explaining the different findings with respect to evolution of fatigue over time.

NiQoLe revealed a considerable discrepancy between patient-reported and physician-reported AEs. The most common physician-reported AEs were hematological effects (thrombocytopenia, anemia), fatigue/asthenia, and low-grade gastrointestinal effects, consistent with data from 5 previous prospective clinical trials.^9^ By definition, the PRO-CTCAE focuses on symptomatic AEs (eg, fatigue, nausea, insomnia, constipation, and dry mouth), which were reported at up to 10-fold higher incidences by patients compared with physicians. This discordance suggests that clinicians may not report AEs that are most bothersome to patients. The PRO-CTCAE is different from and complementary to the CTCAE,^15^ and the expectations of patients and physicians may differ, highlighting the importance of assessing both to fully understand the impact of treatment on patients. Surveys suggest a disconnect between physician and patient perceptions of AEs,^30^ and patients may be reluctant to report low-grade AEs.^31^ PRO-CTCAEs shed light on lower-grade AEs that may escalate to more severe toxicity or lead to poor treatment compliance or discontinuation because of their cumulative impact on HRQoL. Physicians accessing real-time information to modify treatment may have a more immediate impact on treatment burden. However, there is often reluctance to integrate PRO results into clinical practice.^32^ Clinical staff need to be motivated to use patient-reported tolerability to identify symptoms and initiate supportive measures. Furthermore, prospectively collected data from longitudinal real-world studies are needed to provide complementary data to support findings from pivotal trials, extend our understanding of the treatment burden to less-selected populations presenting in everyday clinical practice, and enable the development of more generalized strategies to monitor and manage side effects of new drugs in broader populations.

NiQoLe enrolled a poor-prognosis population of older patients (54% aged >70 years vs 17% aged ≥70 years in NOVA^33^) predominantly with *BRCA*-wildtype disease, reflecting widespread use of olaparib for patients with *BRCA*-mutated OC in France. Only 35% had a complete response following previous platinum (vs ≥50% in NOVA^3^ and NORA^7^). These less favorable characteristics may explain the shorter-than-expected PFS (median 6 months vs 9 months in the NOVA non-*BRCA*-mutated population^3^).

A weakness of the NiQoLe study is the focus on collecting self-reported AEs without proactive real-time monitoring to improve treatment tolerability. Furthermore, this first-generation device had no alert to patients and/or physicians. Studies since the start of the COVID-19 pandemic have explored how telemedicine can help to manage toxicities. Arriola et al. reported high adoption and adherence to weekly symptom monitoring via personal devices and improved interactions and care.^34^ Another study demonstrated the feasibility, acceptability, and preliminary efficacy of a telehealth intervention in reducing the interference and severity of fatigue during PARPi therapy for advanced OC.^35^

Given the feasibility and logistical simplicity of the digital tool used in NiQoLe, we suggest that future trials of PARPis and novel investigational agents should integrate the PRO-CTCAE into regular follow-up. Real-world studies should incorporate both patient perspectives and standard physician-reported safety monitoring to allow more comprehensive assessment and management of side effects, treatment burden, and impact on HRQoL, enabling better management of toxicity. Furthermore, these tools could be used in routine practice to minimize toxicity and increase the feasibility of maintenance therapy.

## Supplementary Material

pkae114_Supplementary_Data

## Data Availability

Currently, no mechanism is in place to allow sharing of individual deidentified patient data. Requests sent to ARCAGY-GINECO (sarmanet@arcagy.org) will be considered on a case-by-case basis.
