# Supplementary material for: Improving real-world evaluation of patient- and physician-reported tolerability: niraparib for recurrent ovarian cancer (NiQoLe)
Source: JNCI Cancer Spectr. 2024 Dec 14;9(1):pkae114. doi: 10.1093/jncics/pkae114 (PMC11878563; doi:10.1093/jncics/pkae114)

Supplementary Materials

Supplementary Methods. PRO-CTCAEs

The Patient-Reported Outcomes version of the Common Terminology Criteria for Adverse Events (PRO-CTCAE) combines individual scores for frequency, severity, and interference with daily activities for each adverse event (Supplementary Table 1) into a composite numeric grade ranging from 0 to 3.[1]

Supplementary Table 1. Scoring Grades for the Frequency, Severity, Interference, and Presence/Absence of Adverse Events

	**0**	**1**	**2**	**3**	**4**
**Frequency**	Never	Rarely	Occasionally	Frequently	Almost constantly
**Severity**	None	Mild	Moderate	Severe	Very severe
**Interference**	Not at all	A little bit	Somewhat	Quite a bit	Very much
**Presence/ absence**	Absent	Present	N/A	N/A	N/A

Abbreviation: N/A, not applicable.

The most relevant items of the PRO-CTCAE selected for evaluation in the NiQoLe study are shown in Supplementary Table 2.

Supplementary Table 2. PRO-CTCAE Items Selected for Inclusion in NiQoLe

**Gastrointestinal**	**Oral**	**Neurological**	**Sleep/wake**
Taste changes (S)	Dry mouth (S)	Dizziness (SI)	Insomnia (SI)
Decreased appetite (SI)	Mouth/throat sores (SI)	**Attention/memory**	Fatigue (SI)
Nausea (FS)	**Cardio/circulatory**	Concentration (SI)	**Mood**
Vomiting (FS)	Heart palpitations (FS)	Memory (SI)	Anxious (FSI)
Constipation (S)	**Cutaneous**	**Pain**	Discouraged (FSI)
Abdominal pain (FSI)	Rash (P)	Headache (FSI)	

Abbreviations: F, frequency; I, interference; P, presence/absence; S, severity.

Reference

1. Basch E, Becker C, Rogak LJ, et al. Composite grading algorithm for the National Cancer Institute's Patient-Reported Outcomes version of the Common Terminology Criteria for Adverse Events (PRO-CTCAE). Clin Trials 2021;18:104–114. https://doi.org/10.1177/1740774520975120

Supplementary Figure 1. NiQoLe study weekly electronic reporting of patient-reported outcomes via a CHES. The CHES platform is a specialized software dedicated to the assessment, storage, and processing of electronically collected patient-reported outcome data from EORTC questionnaires.[1]

Abbreviations: CHES, computer-based health evaluation system; EORTC, European Organisation for Research and Treatment of Cancer; FACT-F, Functional Assessment of Cancer Therapy-Fatigue; PRO-CTCAE, Patient-Reported Outcome version of the Common Terminology Criteria for Adverse Events.

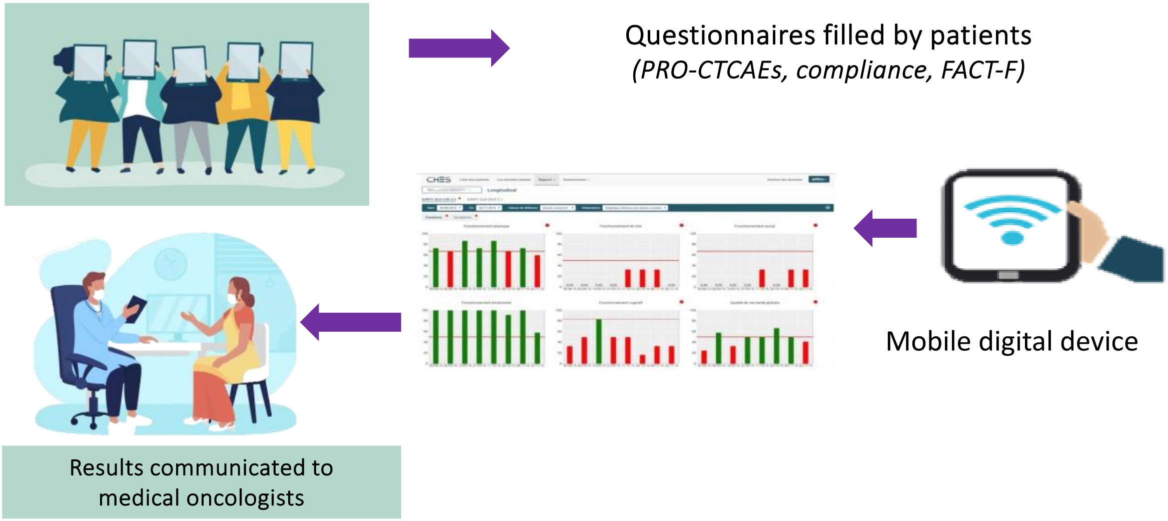

Questionnaires were completed on paper instead of electronically by 29 patients.

Reference

1. EORTC. CHES platform. Available at: https://qol.eortc.org/ches-platform/#1 (accessed May 10, 2024).

Supplementary Figure 2. Swimmer plot of patients with ≥1 treatment modification for an AE during the first 3 months.

Abbreviation: AE, adverse event.

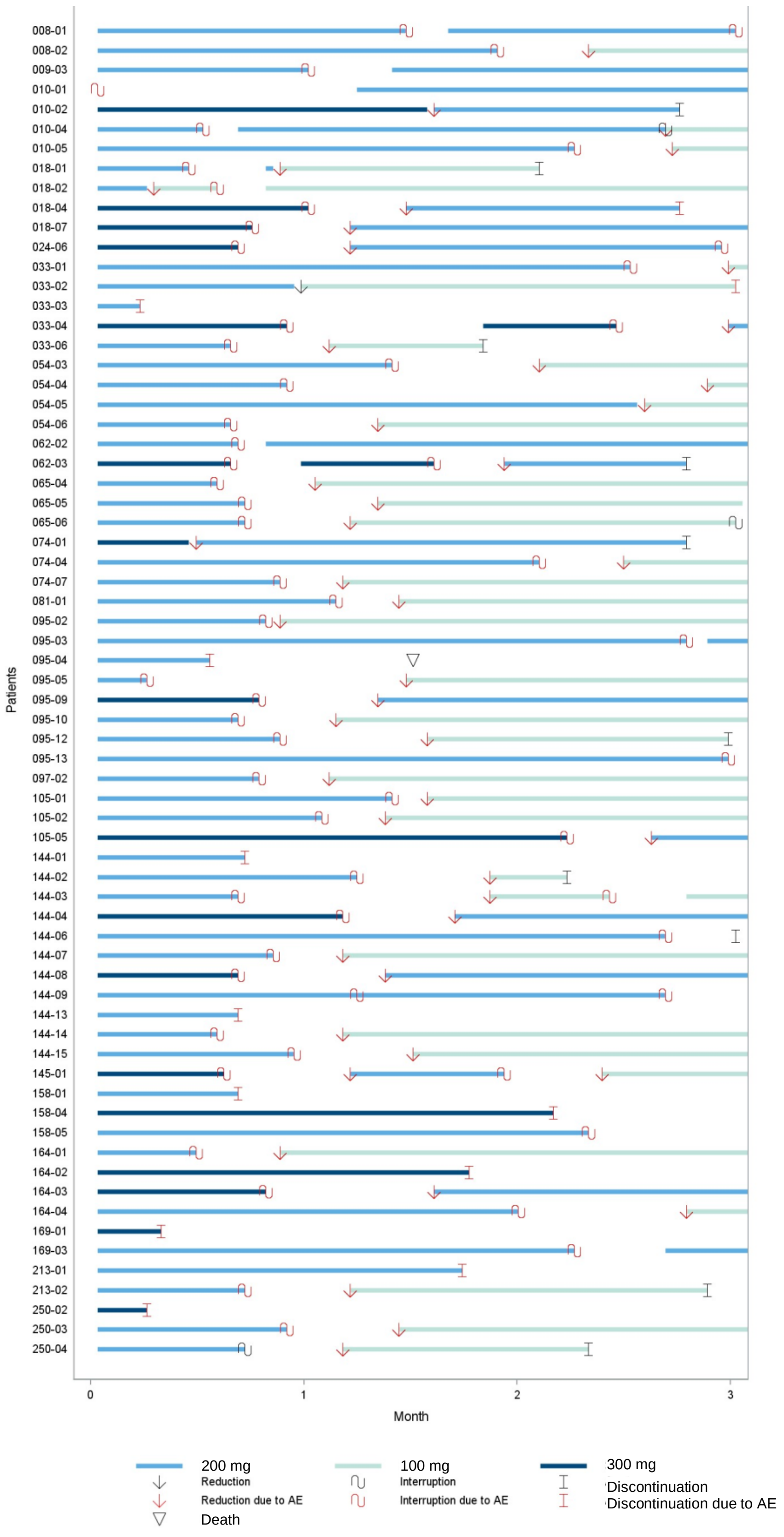
Patients
Month
0
1
2
3
200 mg
100 mg
300 mg
Reduction
Interruption
Discontinuation
Reduction due to AE
Interruption due to AE
Discontinuation due to AE
Death
008-01
008-02
009-03
010-01
010-02
010-04
010-05
018-01
018-02
018-04
018-07
024-06
033-01
033-02
033-03
033-04
033-06
054-03
054-04
054-05
054-06
062-02
062-03
065-04
065-05
065-06
074-01
074-04
074-07
081-01
095-02
095-03
095-04
095-05
095-09
095-10
095-12
095-13
097-02
105-01
105-02
105-05
144-01
144-02
144-03
144-04
144-06
144-07
144-08
144-09
144-13
144-14
144-15
145-01
158-01
158-04
158-05
164-01
164-02
164-03
164-04
169-01
169-03
213-01
213-02
250-02
250-03
250-04

Supplementary Figure 3. Completion rate for PRO-CTCAE, treatment compliance, and FACT-F questionnaires at each timepoint (as a percentage of the number expected to complete, ie, patients still on study).

Abbreviations: FACT-F, Functional Assessment of Cancer Therapy-Fatigue; PRO-CTCAE, Patient-Reported Outcome version of the Common Terminology Criteria for Adverse Events.

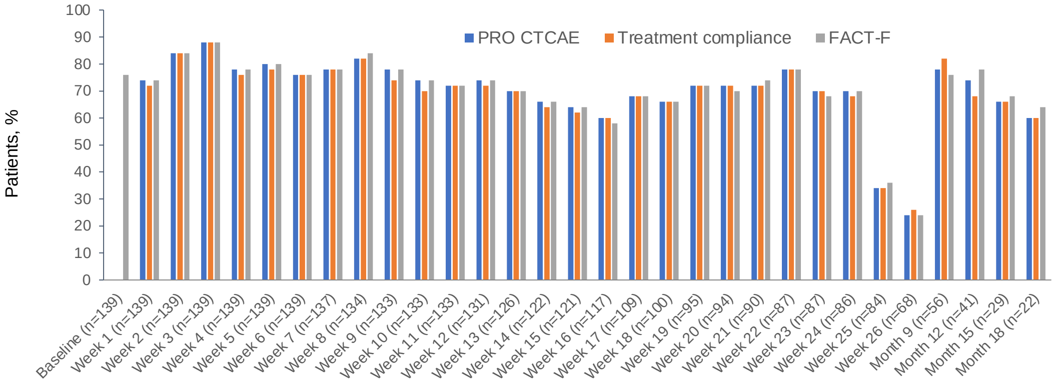

Supplementary Figure 4. Distribution of PRO-CTCAE item groups weekly until week 12 and maximum score post-baseline. **(A)** Insomnia (including difficulty falling asleep, staying asleep, or waking up early). **(B)** Constipation. **(C)** Dry mouth. **(D)** Pain in the abdomen. **(E)** Anxiety. **(F)** Problems with concentration. **(G)** Dizziness. **(H)** Feeling discouraged. **(I)** Headache. **(J)** Problems with memory. **(K)** Mouth or throat sores. **(L)** Pounding or racing heartbeat (palpitations). **(M)** Problems with tasting food or drink (taste changes).

PRO-CTCAE, Patient-Reported Outcome version of the Common Terminology Criteria for Adverse Events.

*Maximum score or grade reported post-baseline per patient.

A) Insomnia

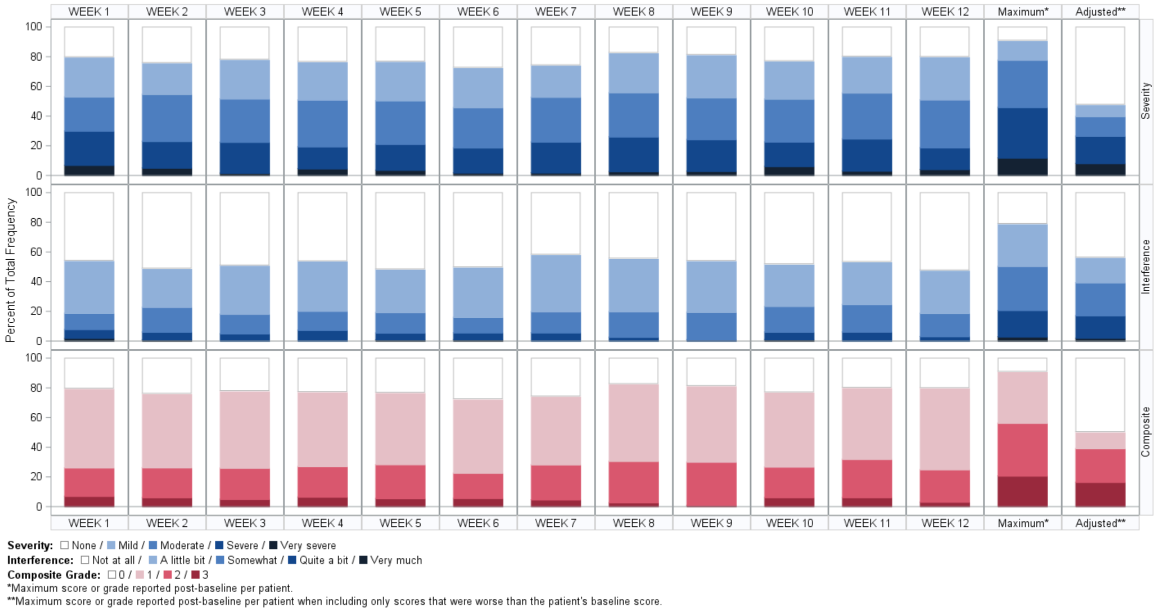

B) Constipation

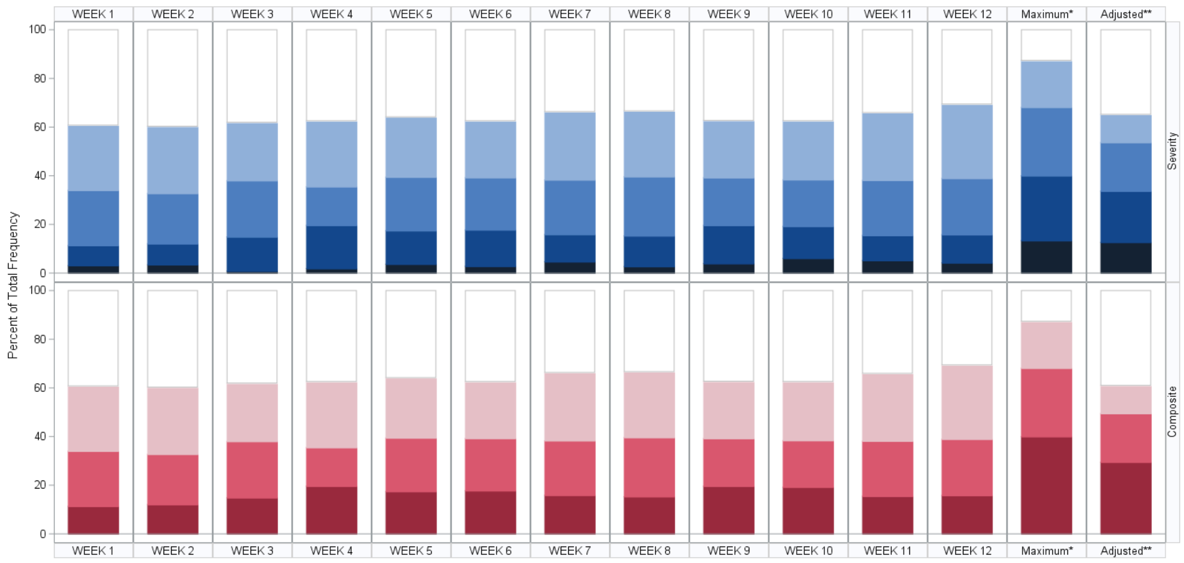

Severity: ☐ None / Mild / Moderate / Severe / Very severe
Composite Grade: ☐ 0 / 1 / 2 / 3
*Maximum score or grade reported post-baseline per patient.
**Maximum score or grade reported post-baseline per patient when including only scores that were worse than the patient's baseline score.

C) Dry mouth

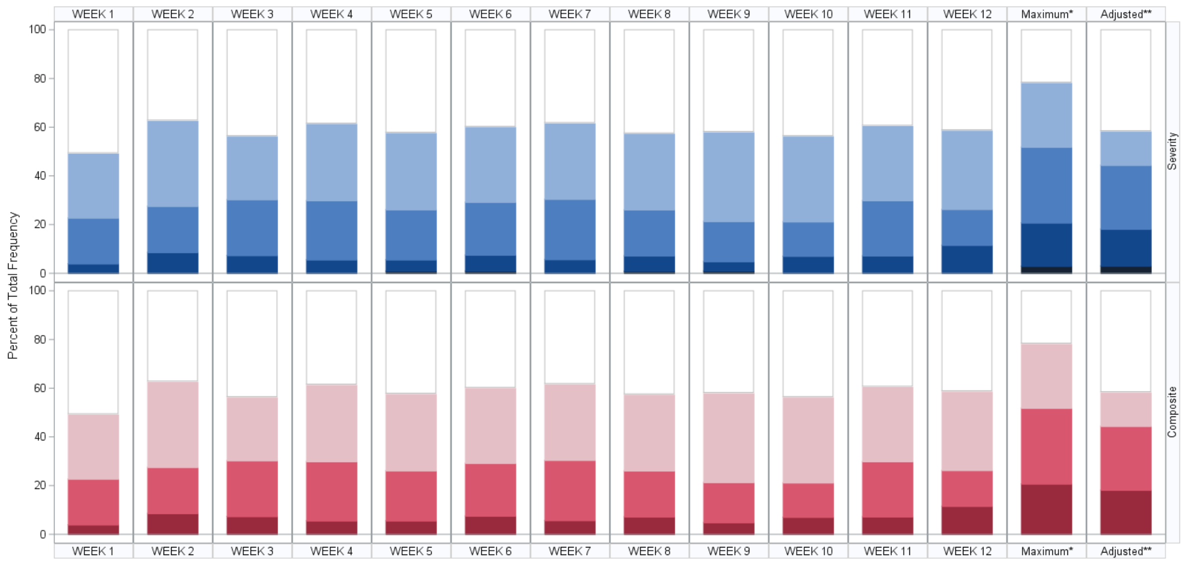

Severity: ☐ None / Mild / Moderate / Severe / Very severe
Composite Grade: ☐ 0 / 1 / 2 / 3
*Maximum score or grade reported post-baseline per patient.
**Maximum score or grade reported post-baseline per patient when including only scores that were worse than the patient's baseline score.

D) Pain in the abdomen

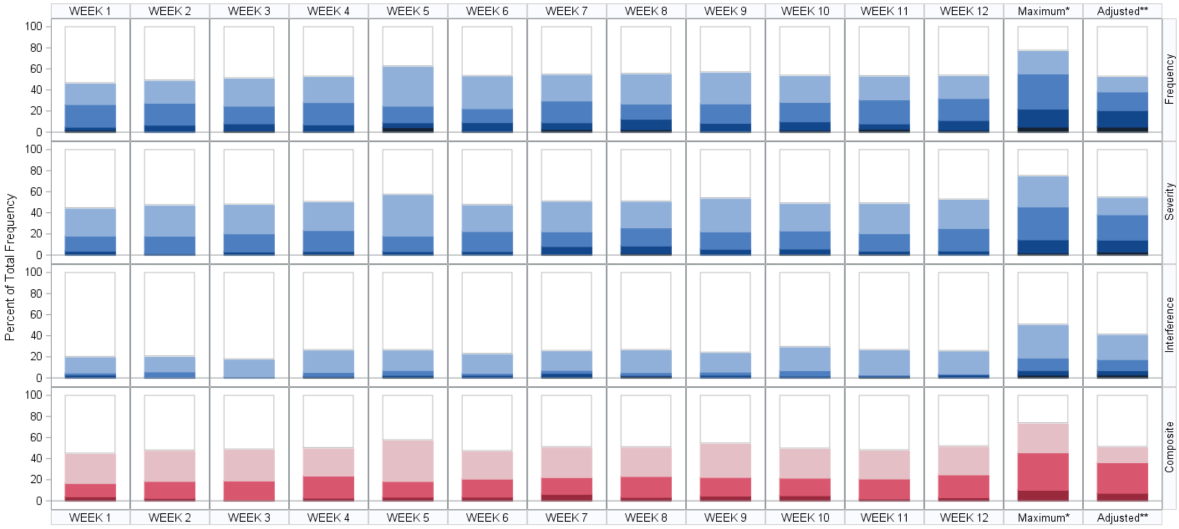

Frequency: ☐ Never / Rarely / Occasionally / Frequently / Almost constantly
Severity: ☐ None / Mild / Moderate / Severe / Very severe
Interference: ☐ Not at all / A little bit / Somewhat / Quite a bit / Very much
Composite Grade: ☐ 0 / 1 / 2 / 3
*Maximum score or grade reported post-baseline per patient.
**Maximum score or grade reported post-baseline per patient when including only scores that were worse than the patient's baseline score.

E) Anxiety

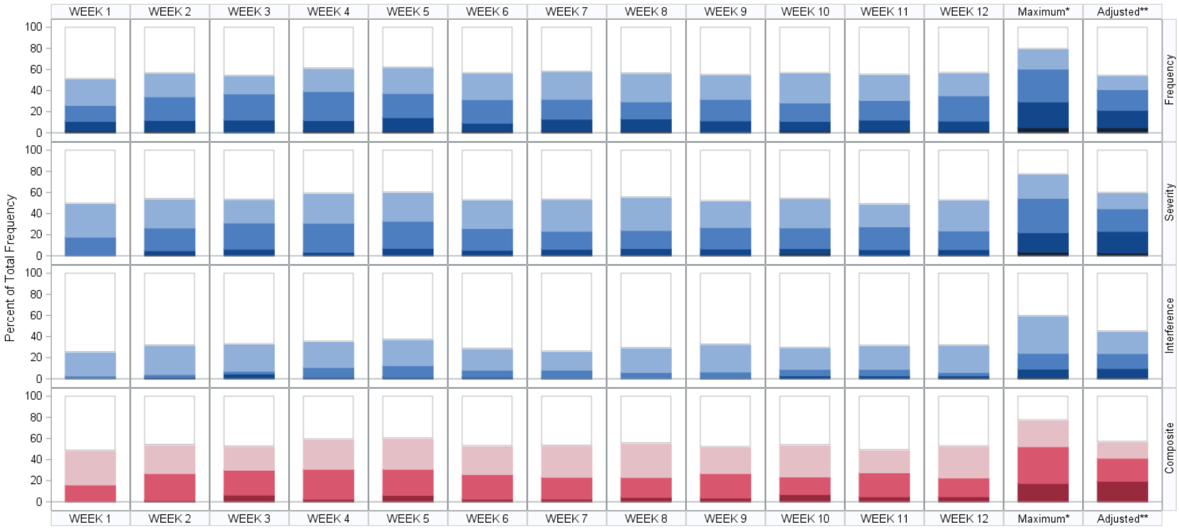

Frequency: ☐ Never / Rarely / Occasionally / Frequently / Almost constantly
Severity: ☐ None / Mild / Moderate / Severe / Very severe
Interference: ☐ Not at all / A little bit / Somewhat / Quite a bit / Very much
Composite Grade: ☐ 0 / 1 / 2 / 3
*Maximum score or grade reported post-baseline per patient.
**Maximum score or grade reported post-baseline per patient when including only scores that were worse than the patient's baseline score.

F) Problems with concentration

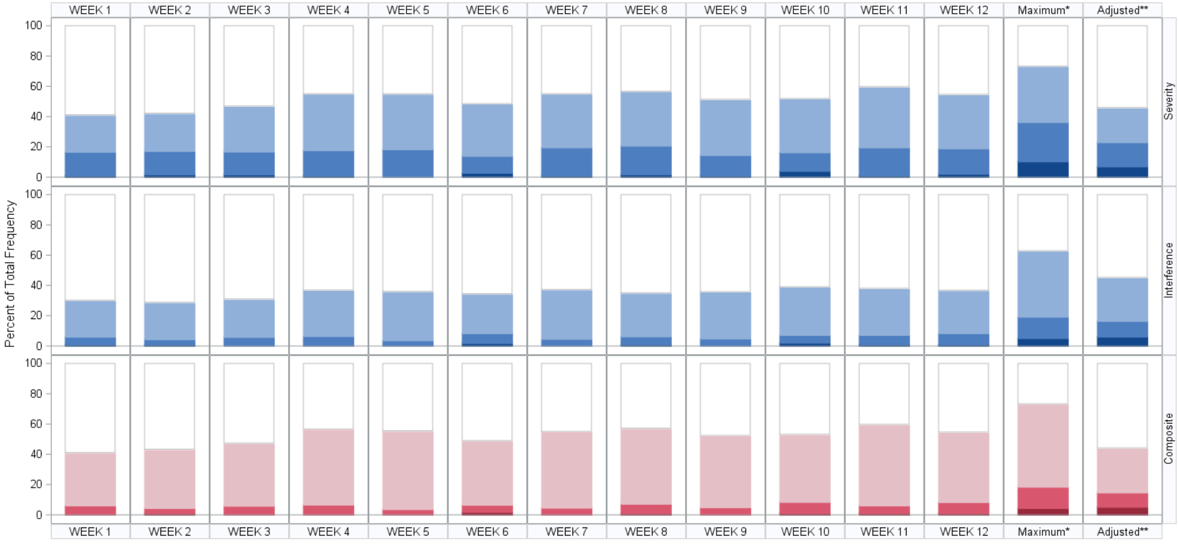

Severity: ☐ None / Mild / Moderate / Severe / Very severe
Interference: ☐ Not at all / A little bit / Somewhat / Quite a bit / Very much
Composite Grade: ☐ 0 / 1 / 2 / 3
*Maximum score or grade reported post-baseline per patient.
**Maximum score or grade reported post-baseline per patient when including only scores that were worse than the patient's baseline score.

G) Dizziness

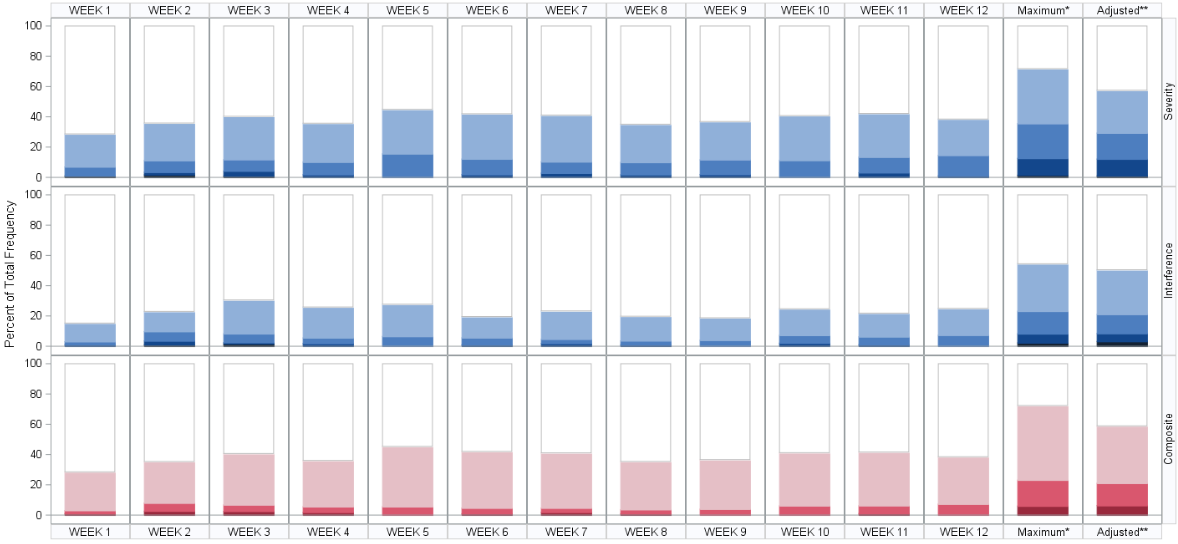

Severity: ☐ None / Mild / Moderate / Severe / Very severe
Interference: ☐ Not at all / A little bit / Somewhat / Quite a bit / Very much
Composite Grade: ☐ 0 / 1 / 2 / 3
*Maximum score or grade reported post-baseline per patient.
**Maximum score or grade reported post-baseline per patient when including only scores that were worse than the patient's baseline score.

H) Feeling discouraged

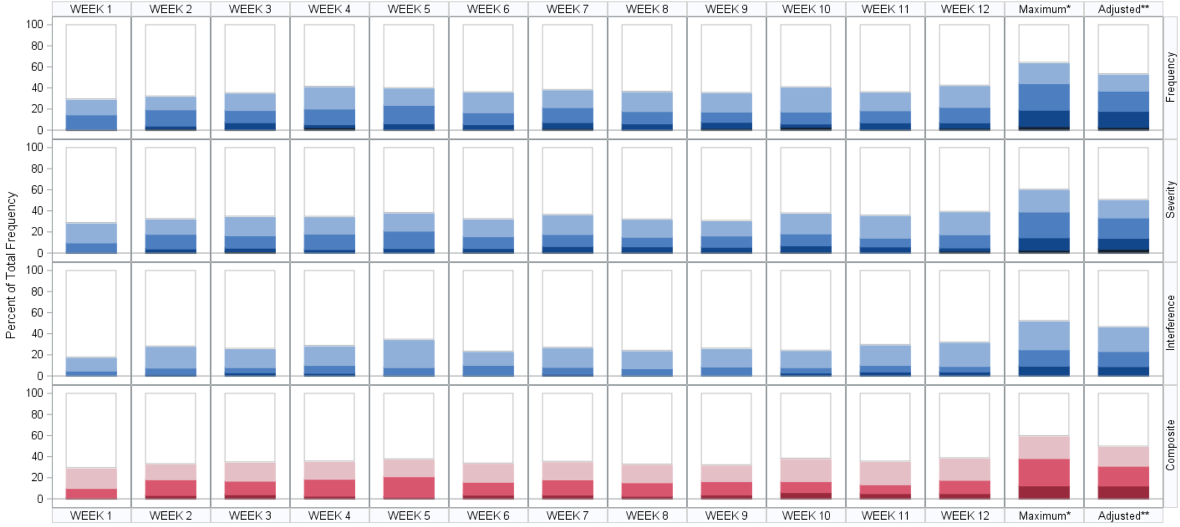

Frequency: ☐ Never / Rarely / Occasionally / Frequently / Almost constantly
Severity: ☐ None / Mild / Moderate / Severe / Very severe
Interference: ☐ Not at all / A little bit / Somewhat / Quite a bit / Very much
Composite Grade: ☐ 0 / 1 / 2 / 3
*Maximum score or grade reported post-baseline per patient.
**Maximum score or grade reported post-baseline per patient when including only scores that were worse than the patient's baseline score.

I) Headache

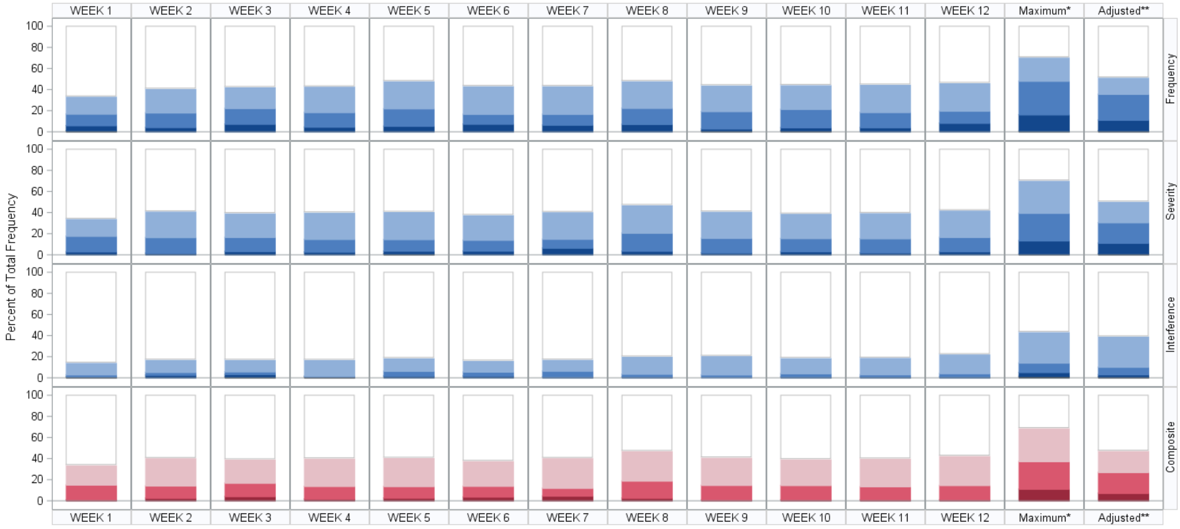

Frequency: ☐ Never / Rarely / Occasionally / Frequently / Almost constantly
Severity: ☐ None / Mild / Moderate / Severe / Very severe
Interference: ☐ Not at all / A little bit / Somewhat / Quite a bit / Very much
Composite Grade: ☐ 0 / 1 / 2 / 3
*Maximum score or grade reported post-baseline per patient.
**Maximum score or grade reported post-baseline per patient when including only scores that were worse than the patient's baseline score.

J) Problems with memory

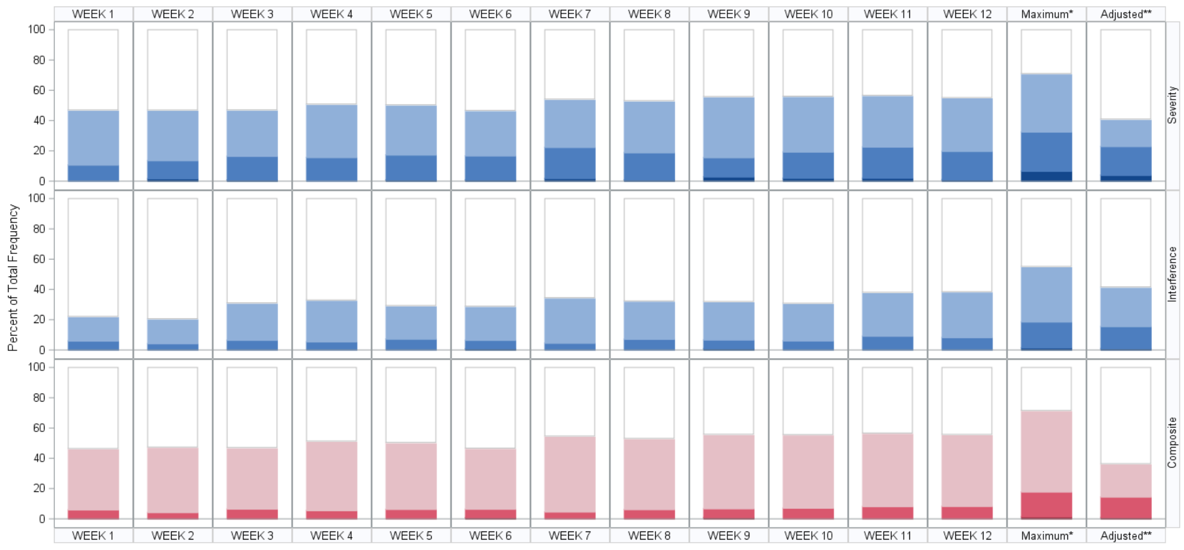

Severity: ☐ None / Mild / Moderate / Severe / Very severe
Interference: ☐ Not at all / A little bit / Somewhat / Quite a bit / Very much
Composite Grade: ☐ 0 / 1 / 2 / 3
*Maximum score or grade reported post-baseline per patient.
**Maximum score or grade reported post-baseline per patient when including only scores that were worse than the patient's baseline score.

K) Mouth or throat sores

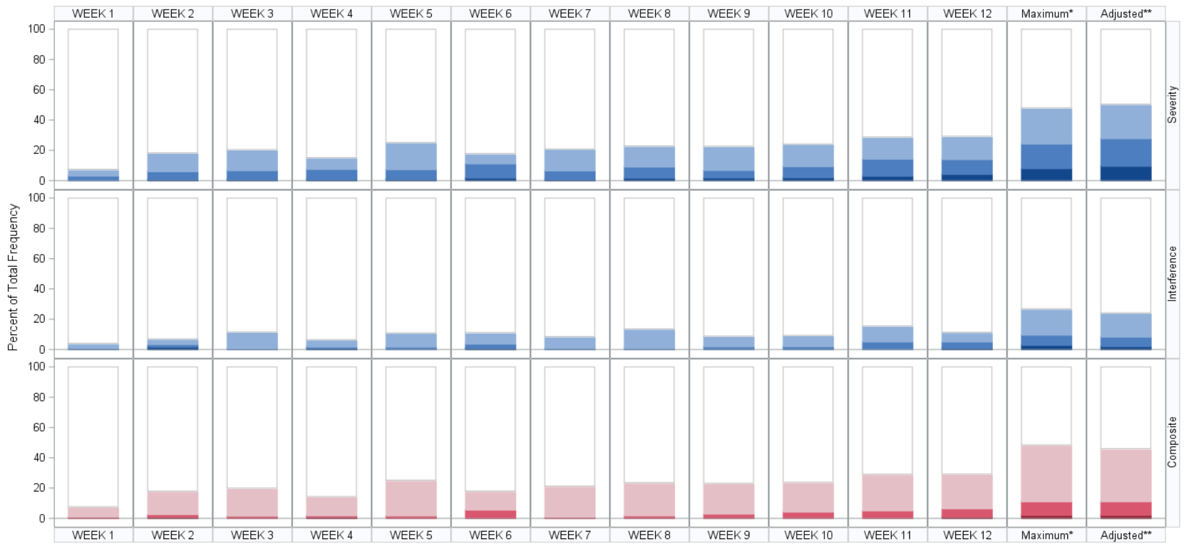

Severity: ☐ None / Mild / Moderate / Severe / Very severe
Interference: ☐ Not at all / A little bit / Somewhat / Quite a bit / Very much
Composite Grade: ☐ 0 / 1 / 2 / 3
*Maximum score or grade reported post-baseline per patient.
**Maximum score or grade reported post-baseline per patient when including only scores that were worse than the patient's baseline score.

L) Pounding or racing heartbeat (palpitations)

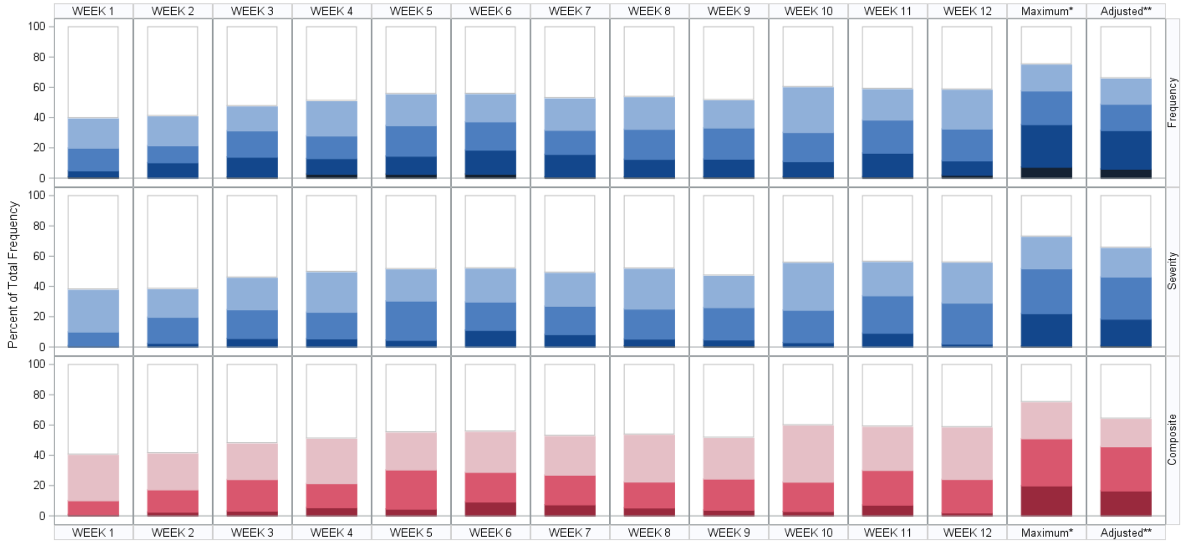

Frequency: ☐ Never / ■ Rarely / ■ Occasionally / ■ Frequently / ■ Almost constantly
Severity: ☐ None / ■ Mild / ■ Moderate / ■ Severe / ■ Very severe
Composite Grade: ☐ 0 / ■ 1 / ■ 2 / ■ 3
*Maximum score or grade reported post-baseline per patient.
**Maximum score or grade reported post-baseline per patient when including only scores that were worse than the patient's baseline score.

M) Problems with tasting food or drink

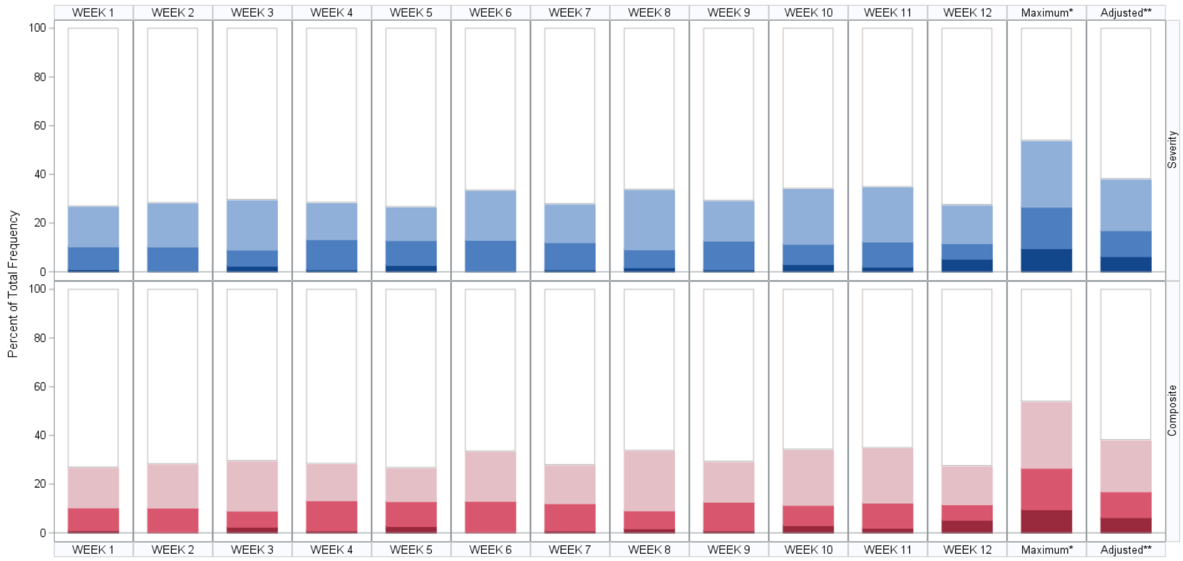

Severity: ☐ None / ■ Mild / ■ Moderate / ■ Severe / ■ Very severe
Composite Grade: ☐ 0 / ■ 1 / ■ 2 / ■ 3
*Maximum score or grade reported post-baseline per patient.
**Maximum score or grade reported post-baseline per patient when including only scores that were worse than the patient's baseline score.

Supplementary Figure 5. FACT-F fatigue scores. **(A)** Mean FACT-F score over time in patients with a baseline score. Error bars represent standard deviation. **(B)** Time to worsening of FACT-F score in patients with a baseline score.

Abbreviations: CI, confidence interval; FACT-F, Functional Assessment of Cancer Therapy-Fatigue.

A

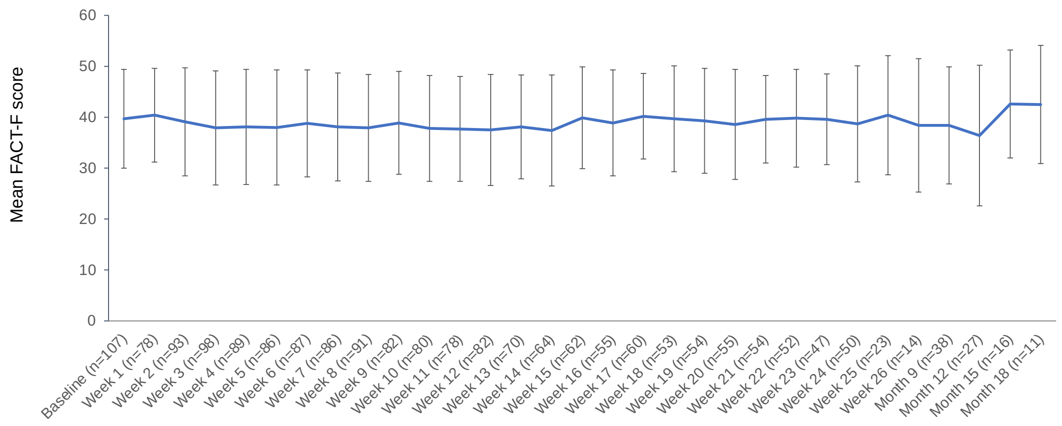

B

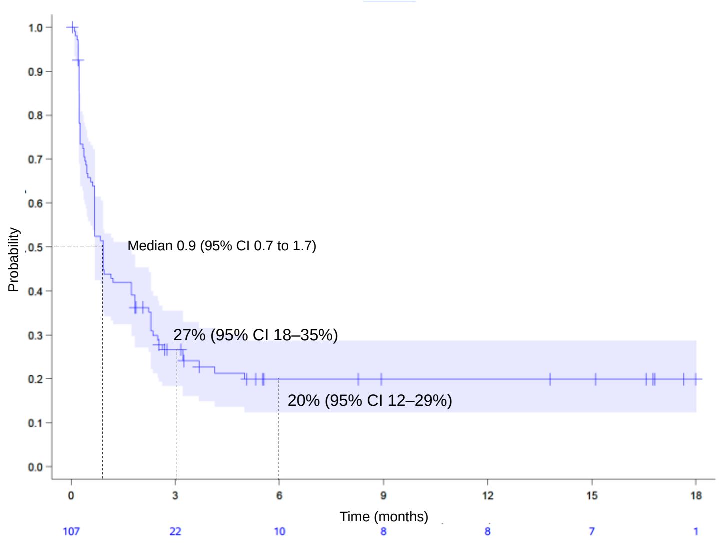

Supplement: pkae114_Supplementary_Data [file pkae114_supplementary_data.pdf]
